# The ground-level ozone concentration is inversely correlated with the number of COVID-19 cases in Warsaw, Poland

**DOI:** 10.1007/s11869-021-01009-7

**Published:** 2021-04-08

**Authors:** Oskar Wiśniewski, Wiesław Kozak, Maciej Wiśniewski

**Affiliations:** 1 Department of Immunology, Nicolaus Copernicus University in Toruń, 1 Lwowska Street, Toruń, Poland; 2 ImmQuest, 17 Uniwersytecka Street, Toruń, Poland

**Keywords:** Coronavirus, SARS-CoV-2, Poland, Weather, Ozone

## Abstract

COVID-19, which is a consequence of infection with the novel viral agent SARS-CoV-2, first identified in China (Hubei Province), has been declared a pandemic by the WHO. As of September 10, 2020, over 70,000 cases and over 2000 deaths have been recorded in Poland. Of the many factors contributing to the level of transmission of the virus, the weather appears to be significant. In this work, we analyze the impact of weather factors such as temperature, relative humidity, wind speed, and ground-level ozone concentration on the number of COVID-19 cases in Warsaw, Poland. The obtained results show an inverse correlation between ground-level ozone concentration and the daily number of COVID-19 cases.

## Introduction

COVID-19 is a disease caused by the novel viral agent SARS-CoV-2. It was first identified and described in central China (Wuhan, Hubei Province) in December 2019. The World Health Organization announced COVID-19 outbreak a pandemic on March 12, 2020. Until now (September 10, 2020), over 27 million cases and nearly 900,000 deaths caused by this disease were reported worldwide. In Poland, these rates amounted to over 70,000 cases and over 2000 deaths. Each COVID-19 case follows an individual course. Most infected people develop symptoms that can be described as mild to moderate. Such people recover without hospitalization. However, severe symptoms of the disease are shortness of breath, high fever, cough, loss of sense of smell, and muscle pain. The mean incubation period of SARS-CoV-2 was estimated to be 6.4 days, ranging from 2.1 to 11.1 days (Lauer et al. 2020).

The first case of the novel coronavirus in Poland was reported on March 4, 2020 (in the western region of the country). The Polish government declared the state of epidemic threat on March 14, 2020, and the state of epidemic was declared on March 20, 2020. At this time, many restrictions were introduced, such as the obligation to wear face masks, a ban on gathering and moving in groups, and the closure of certain service points. It was also recommended not to travel for Easter and the long weekend (in the beginning of May).

It is widely known that weather conditions can influence the transmission of various viruses. Bi et al. (2007) found an inverse association between the number of daily cases of SARS and ambient temperature, and a positive association with air pressure during the epidemic of 2003 in Beijing and Hong Kong. Moreover, several epidemiological analyses identified absolute humidity and temperature as climatic predictors of influenza virus transmission in temperate regions of the world (Lowen and Steel 2014). The mechanisms by which weather factors shaping the viral transmissibility remain unclear. Possible explanations consider the instability of some viruses at high temperatures, better adhesion to objects at higher humidity, and higher transmission during strong winds.

In the present report, we analyze the impact of daily average temperature, relative humidity, wind speed, and ground-level ozone concentration on the number of daily COVID-19 cases. As far as we know, our work is the first to consider the impact of weather factors on SARS-CoV-2 transmission in Poland.

## Methods

### Study area

Warsaw is the capital of the Republic of Poland. It is located in the central-eastern part of the country (52° 14′ 13.3764″ N, 21° 1′ 3.1152″ E), in the area of humid continental climate (Köppen climate classification). According to data from December 31, 2019, it is inhabited by 1,790,658 people and its area covers 517.24 km^2^ (including the Vistula river).

### Data collection

Data on the daily number of cases (confirmed by the RT-PCR method) between April 7, 2020, and June 7, 2020, were obtained from the official reports of the District Sanitary-Epidemiological Station in Warsaw. The weather data from April 1, 2020, to June 1, 2020: daily average temperature [°C], relative humidity [%], and wind speed [m/s] for the period of 2 months were obtained from the publicly available repository of Polish Institute of Meteorology and Water Management (*Warszawa-Bielany* weather monitoring station). The daily ground-level ozone (O_3_) [μg/m^3^] measurements were obtained from the publicly available database of the Chief Inspectorate of Environmental Protection (*Warszawa-Chroscickiego* air pollution monitoring station). Data on confirmed daily cases were paired with weather data 7 days earlier (due to reasons described below).

### Data analysis

Since the data were not normally distributed, the Spearman rank correlation test was used to examine the relationship between particular weather factors and daily COVID-19 cases.

## Results and discussion

**Fig. 1:**
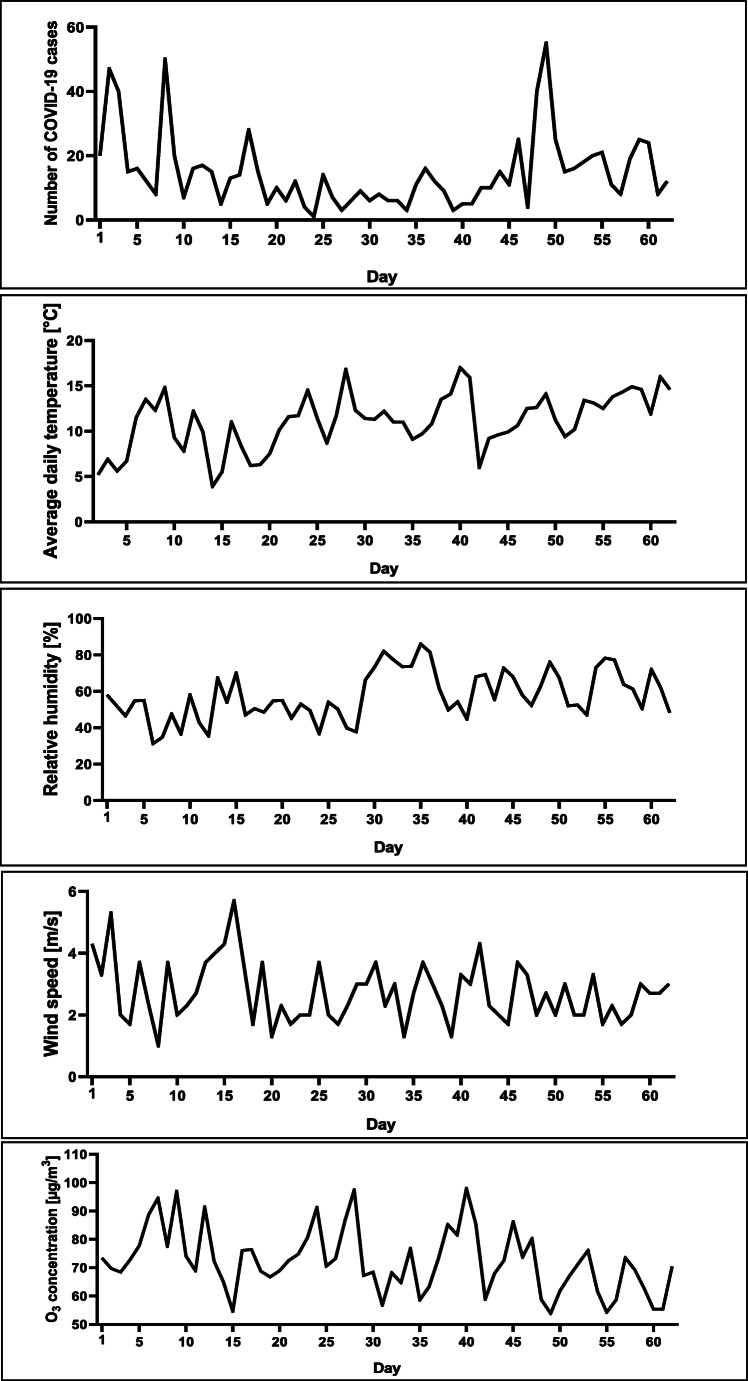
Daily cases of the COVID-19, daily average temperature [°C], relative humidity [%], wind speed [m/s], and average ground-level zone concentration [μg/m^3^] in Warsaw, Poland, from April 7 to June 7, 2020 (number of COVID-19 cases) and April 1 to June 1, 2020 (weather variables)

Between April 7, 2020, and June 7, 2020, a total number of 917 COVID-19 cases were reported in Warsaw. The lowest daily average temperature recorded in the period between April 1, 2020, and June 1, 2020, was 3.9 °C, while the highest daily average temperature was 20.1 °C. The lowest daily relative humidity recorded was 35.5%, while the highest daily relative humidity was 87%. The lowest wind speed recorded was 1 m/s, while the highest wind speed was 5.7 m/s. The lowest daily average ground-level O_3_ concentration recorded was 53.9 μg/m^3^, while the highest ground-level O_3_ concentration was 97.9 μg/m^3^. Changes of respective values over time are presented in Fig. 1.

The obtained data indicate that only the average daily ambient ozone concentration is inversely correlated with the number of COVID-19 cases (Table 1). Pairing of the daily case numbers with the weather conditions 7 days earlier is in our opinion corresponds more to reality than comparing the case numbers and the weather conditions on the same day. It was done due to the average incubation period of the coronavirus and the average 24-h waiting period for the RT-PCR test result in Poland.

**Table 1:** Spearman correlation coefficients (*ρ*) and their *P*-values between COVID-19 cases and weather variables

Weather variable	Spearman’s *ρ*	*P*-value
Average daily temperature [°C]	− 0.1195	0.3589
Relative humidity [%]	0.0389	0.7636
Wind speed [m/s]	0.1355	0.2937
Ground-level ozone concentration [μg/m^3^]	− 0.2997*	0.0180

*Correlation is significant at the 0.05 level (two-tailed)

There is a number of publications describing the impact of weather conditions on the spread of the novel coronavirus. Tosepu et al. (2020) found that only daily average temperature was significantly correlated with number of COVID-19 cases in Jakarta. Luo et al. (2020) reported a positive correlation between absolute humidity and case increase, and a weak negative correlation between weather temperature and case increase in several regions in Asia. A study on the effects of temperature and humidity on the daily new cases and new deaths of COVID-19 in 166 countries revealed that both factors were negatively related to the daily new cases and daily new deaths of COVID-19 (Wu et al. 2020). Poirier et al. (2020) indicated that higher temperatures and locations with higher absolute humidity appeared to have lower COVID-19 transmission. The authors have also identified potential confounders, suggesting that changes in weather alone may not lead to changes in case count without the implementation of public health interventions. Jiwei et al. (2020) proposed that a decrease in the transmissibility of COVID-19 is likely with the arrival of spring in the north hemisphere. However, temperature and humidity were found to contribute to a maximum of 18% of the variation, the remaining 82% to social factors. In their study, Bu et al. (2020) found that the suitable temperature range for SARS-CoV-2 survival is 13–24 °C. Unfortunately, the authors have not identified any confounders and potential strategies to deal with them. Shi et al. (2020) in their retrospective observational study concluded that both low and high temperatures might decrease the COVID-19 incidence, but the duration of the outbreak would depend on the safety measures introduced by particular countries. Ran et al. (2020b) found that the ambient temperature has a nonlinear negative association with COVID-19 transmission. It is also remarked that control strategies for the COVID-19 pandemic barely relying on environmental factors are unlikely to succeed. On the other hand, Yao et al. (2020) showed no association of COVID-19 transmission with temperature or UV radiation in China.

Only a few papers describe the effects of ambient ozone concentration on COVID-19. A recent paper from China shows that the COVID-19 transmissibility could be negatively associated with ambient ozone (Ran et al. 2020a). On the contrary, a positive correlation was found between COVID-19 infections and ground-level ozone in Milan, Italy (Zoran et al. 2020). An analysis performed by Adhikari and Yin (2020) showed that daily average temperature, daily maximum 8-h ozone concentration, average relative humidity, and cloud percentages were significantly and positively associated with new confirmed cases related to COVID-19, but none of these variables showed significant associations with new deaths related to COVID-19.

Ozone is generally considered an antiviral agent that damages the viral capsid and upsets its reproductive cycle (Elvis and Ekta 2011). However, a review of the literature shows that ozone doses that have a virucidal effect range from 0.6 to 6.0 ppm for 20–112 min (Tseng and Li 2008; Yano et al. 2020). Commercial ozone generators guaranteed antiviral efficacy at 10–20 ppm for 10 min (Dennis et al. 2020; Grignani et al. 2021). It is therefore clear that the ambient ozone levels are several orders of magnitude lower than the doses used to eliminate respiratory viruses. However, we propose a mechanism that may explain the inverse correlation described above based on alterations of immune system functions. There are a number of reports suggesting that both innate and acquired immunity are regulated by ambient ozone. In general, environmental ozone modifies cell types required for priming innate immunity and contributes to innate-adaptive immune system cross-talk (Al-Hegelan et al. 2011). The alterations are visible both in the number and type of cells of the immune system and in the substances produced by these cells. For example, increased level of ambient ozone was found to activate innate immune signaling in airway epithelia (Gribar et al. 2008) and to suppress T-lymphocyte-dependent immune response (Al-Hegelan et al. 2011). Moreover, changes in production of proteins involved in immune signaling (interleukin-1, interleukin-6, interleukin-17, and tumor necrosis factor α) were observed (Al-Hegelan et al. 2011). We propose that the activity of another cytokine, interleukin-33, could be a potential explanation of the observed inverse correlation. According to the literature, interleukin-33 is induced by ozone and further activates Th2 phenotype cytokines in the respiratory tract (Ali et al. 2018). On the other hand, there is an emerging evidence that Th2-skewed immunity might be protective in COVID-19 patients. This phenotype is able to downregulate the late phase of disease which is typically associated with uncontrolled inflammation (Carli et al. 2020). The protective role of Th2 phenotype is probably mainly due to the eosinophils. Increase in the number and activation of these cells have traditionally been linked to allergies and asthma, but it turns out that eosinophils, besides their proinflammatory effects, play a crucial role in antiviral responses (Franceschini et al. 2020). Our candidate, interleukin-33, regulates eosinophils at three stages: development within the bone narrow, activation of matured cells, and activation of mature eosinophils within the tissue (Johnston and Bryce 2017). Therefore, we suggest that the increased activity of interleukin-33 under the influence of ambient ozone may be responsible for the creation of an immune microenvironment that protects against SARS-CoV-2 infection.

It must be emphasized that our research has some limitations. Firstly, the number of COVID-19 cases may not be directly related to virus transmission. In the described period, the Polish government did not provide the daily number of tests performed, but only informed about the number of positive cases. Moreover, we suspect that asymptomatic or oligosymptomatic cases may have missed the tests for fear of worsening the quality of life (due to quarantine or domestic isolation). Secondly, confounding variables must be taken into consideration. General health policies for a given period, containment measures, public health interventions, population density, and cultural aspects—all of these can contribute to the spread of the virus. Warsaw, as the largest city in the country, is the main communication hub, and the mobility of its inhabitants is at a high level—many of them are commuters from surrounding regions. The inhabitants are characterized by different hygienic habits, lifestyle, profession, etc. Thus, we believe that weather factors cannot be considered separately from social factors.

## Conclusions

Of the factors studied, only ozone concentration seems to have an impact on the number of new COVID-19 cases. Further research is needed, for example, in other Polish cities, to characterize the remaining weather factors that may influence the spread of the virus.

## Data Availability

The datasets analyzed during the current study are available in the IMGW repository (temperature, humidity, wind speed) (https://www.imgw.pl/en) and GIOS repository (ozone) (http://powietrze.gios.gov.pl/pjp/current).
